# Mapping Interpersonal Emotion Regulation in Everyday Life

**DOI:** 10.1007/s42761-023-00223-z

**Published:** 2023-11-13

**Authors:** Anh Tran, Katharine H. Greenaway, Joanne Kostopoulos, Sarah T. O’Brien, Elise K. Kalokerinos

**Affiliations:** 1 Melbourne School of Psychological Sciences, University of Melbourne, Redmond Barry Building, Parkville, VIC 3010 Australia; 2 School of Psychology, Deakin University, Burwood, Australia

**Keywords:** Interpersonal emotion regulation, Emotion regulation, Daily diary, Experience sampling methodology

## Abstract

The growing literature on interpersonal emotion regulation has largely focused on the strategies people use to regulate. As such, researchers have little understanding of how often people regulate in the first place, what emotion regulation goals they have when they regulate, and how much effort they invest in regulation. To better characterize features of the regulation process, we conducted two studies using daily diary (*N* = 171) and experience sampling methods (*N* = 239), exploring interpersonal emotion regulation in the context of everyday social interactions. We found people regulated others’ emotions nearly twice a day, regulated their own emotions through others around once a day, and regulated *both* their own and others’ emotions in the same interaction roughly every other day. Furthermore, not only did people regulate others’ emotions more often than regulating their own emotions through others, but they also put in more effort to do so. The goals of regulation were primarily to make themselves or others feel better, most often through increasing positive emotions, rather than decreasing negative emotions. Together, these findings provide a foundational picture of the interpersonal emotion regulation landscape, and lay the groundwork for future exploration into this emerging subfield of affective science.

**Supplementary Information:**

The online version contains supplementary material available at 10.1007/s42761-023-00223-z.

The social nature of emotional experiences is at the heart of interpersonal emotion regulation. Interpersonal emotion regulation is delineated into two classes: *intrinsic* (i.e., influencing one’s own emotions through others) and *extrinsic* (i.e., influencing others’ emotions; Zaki & Williams, 2013). Interpersonal emotion regulation research is growing rapidly, resulting in important insights about regulation strategies and outcomes (e.g., Niven et al., 2011; Swerdlow & Johnson, 2022), but there is still little attention on the processes outside of strategy selection and implementation. There is thus limited empirical understanding of how often people regulate in the first place, what emotion regulation goals they have when they regulate, and how much effort they invest. These features are an important part of the regulation process, as they underpin downstream regulation behaviors and outcomes. We aimed to broaden our understanding of these processes by investigating both intrinsic and extrinsic interpersonal emotion regulation in everyday life.

## State of the Literature

Most central to the interpersonal emotion regulation literature is work on regulation strategies. Evidence suggests people make themselves or others feel better through emotional support, cognitive support, acceptance, and social modelling (e.g., Hofmann et al., 2016; Jurkiewicz et al., 2023), or make others feel worse through hostility and inauthentic emotional expression (e.g., Austin & O’Donnell, 2013; Swerdlow & Johnson, 2022). Not only have researchers uncovered a large strategy repertoire (e.g., Niven et al., 2009), they have also shown how strategies are combined and flexibly used in different relationships to optimize outcomes (e.g., Malloch et al., 2020; Battaglini et al., 2023). Thus, the literature has a good understanding of *how* people regulate.

However, by focusing on *how* people regulate, strategy-focused research tacitly assumes people always intend to regulate. Yet, people do not always choose to engage in *intra*personal emotion regulation (e.g., Sheppes, 2020), or indeed *inter*personal emotion regulation (e.g., Liu et al., 2021)—a necessary precursor to strategy selection. As such, to get a complete understanding of interpersonal emotion regulation, it is important to ask a more fundamental question of *how often* people regulate in the first place. To understand the earlier processes that determine whether regulation occurs, we turned to the extended process model of emotion regulation (Gross, 2015).

This model outlines three stages: (a) identification (assessing if regulation is required); (b) selection (choosing a regulation strategy); and (c) implementation (executing the strategy). In the identification stage, people notice whether there is a discrepancy between actual and desired emotions, at which point they may initiate regulation, with an emotion regulation goal (i.e., desired directional change in emotion state) in mind (Tamir, 2021). Having an intention and goal is necessary to initiate strategy selection and implementation (Tamir et al., 2020). Thus, examining intentions (whether people want to regulate) and goals (how they want to feel) is an important step in understanding how and when regulation begins.

A closely related concept is effort, an index of motivational strength reflecting the intensity of regulatory goal pursuit (i.e., how hard people try to regulate) (Gutentag & Tamir, 2022). Leading theoretical frameworks assume that when people intend to regulate, they will put in the effort to do so (Gross, 2015; Zaki & Williams, 2013). However, this is not always the case (e.g., Sheppes, 2020). While specific to *intra*personal emotion regulation, these findings highlight the need to investigate how much effort people invest in regulating *inter*personally. Our understanding of strategy implementation is incomplete without effort; while strategy is the specific regulatory behavior, effort is how intensely people employ that behavior (Tamir, 2021). Knowing whether and how much effort people invest will help refine theoretical models and advance practically relevant research, such as interventions targeting regulation effort.

## Interpersonal Emotion Regulation in Everyday Life

To paint a holistic picture of interpersonal emotion regulation, we must explore the social interactions in which it occurs. Only then will we capture meaningful and consequential emotional experiences (Kuppens et al., 2022). Everyday life techniques, such as daily diary (i.e., sampling participants once a day), and experience sampling methodology (ESM; i.e., sampling participants multiple times per day), are well-poised to achieve this goal. With near-real-time data collection that captures life as it is lived (Bolger et al., 2003), they can provide insights into interpersonal emotion regulation in everyday social interactions.

Liu et al. (2021) were among the first to employ ESM for interpersonal emotion regulation. They found people share negative emotional experiences roughly every other day, most often to seek emotion-oriented and problem-oriented support. However, this work does not explicitly investigate conscious intention to regulate (as sharing negative emotions is not always with a regulation intention; Zaki & Williams, 2013), nor examine regulation effort. Additionally, they studied only intrinsic regulation and negative experiences. Negative experiences have largely been the focus of research, underpinned by the prohedonic assumption that the purpose of emotion regulation is to feel better (Tamir, 2016). This assumption is not entirely unfounded, as people often hold affect-improving goals (Kalokerinos et al., 2017; Springstein et al., 2022). However, theory posits people can also have affect-worsening goals for themselves and others (Niven, 2016; Tamir, 2016), although these are rarely studied. Thus, the research landscape is incomplete, with half the picture missing where (a) extrinsic regulation and (b) affect-worsening goals should be. We aim to provide these missing pieces.

## The Current Research

As existing interpersonal emotion regulation literature has mainly focused on strategy selection and implementation, there is a need to better understand the initial steps underpinning these later stages. We sought to map the features of interpersonal emotion regulation in everyday life, assessing both intrinsic and extrinsic regulation classes to answer three exploratory research questions:

1. How often do people intend to engage in interpersonal emotion regulation?
2. What goals do people hold?
3. How much effort do people invest?

We examined these questions using daily diary (Study 1) to investigate regulation during the most significant social interaction of the day, and ESM (Study 2) to investigate more quotidian social interactions throughout the day. Together, these two studies aimed to provide a more complete picture of everyday interpersonal emotion regulation.

## Study 1

### Method

#### Participants

The sample comprised 171^1^ participants, aged 18 to 62 (*M* = 28.96, *SD* = 11.81, 79% women). Recruitment occurred through an undergraduate research participation program and community advertising. Participants were reimbursed according to compliance with the daily diary protocol (see https://osf.io/ydujv/ for more details).

We used Arend and Schäfer (2019) Monte Carlo simulation-based sensitivity analysis for two-level model to post-hoc determine the minimum detectable effect size. The parameters under the current sample were *N* = 171, seven measurements, and medium-to-large *ICC*s (.30–.50). We aimed for 80% power with *α* = .05. These parameters allowed us to detect a conventionally small effect size of 0.11 (Cohen, 1988).

#### Design and Procedure

The study consisted of three parts: a baseline survey on Day 1, a 7-day daily diary portion, and a follow-up survey on Day 9. All variables of interest were in the daily diaries.

Following the baseline survey, in which participants provided informed consent and completed measures of trait and demographic variables, eligible participants proceeded to the daily diary portion of the study. For seven consecutive days, participants received one diary via Qualtrics to complete any time between 5 pm and 11:59 pm each evening. Each diary asked about participants’ most significant social interaction of the day. A social interaction was defined as, “a verbal exchange (e.g., in person, via phone or video chat) or a written exchange (e.g., social media, text message) with another person that lasted more than 2 min.” If participants did not have any interaction that day, they instead answered questions about a recent significant interaction, which were included for even branching and were not of interest for this study. Participants completed on average six out of seven diaries, yielding a mean compliance rate of 85.96% (*SD* = 22.48) and a total of 1,029 diaries.

#### Measures

##### Intrinsic and Extrinsic Interpersonal Emotion Regulation Intention

Reflecting on their most significant interaction of the day, participants indicated if they tried to (1) “use other people to influence (their) own emotions” (i.e., intrinsic regulation), and (2) “influence the emotions of other people” (i.e., extrinsic regulation).

##### Intrinsic and Extrinsic Interpersonal Emotion Regulation Goals

For both intrinsic and extrinsic regulation, regardless of their intention to regulate, participants could select one or more options to indicate that they (1) had no goal, (2) wanted to increase/maintain own/others’ positive emotions, (3) wanted to decrease own/others’ negative emotions, (4) wanted to increase/maintain own/others’ negative emotions, or (5) wanted to decrease own/others’ positive emotions (adapted from Kalokerinos et al., 2017).

##### Intrinsic and Extrinsic Interpersonal Emotion Regulation Effort

 Regardless of intention to regulate, participants were asked, “How much effort did you put into using other people to influence your own emotions during this interaction?” (i.e., intrinsic regulation), and “How much effort did you put into influencing the emotions of other people during this interaction?” (i.e., extrinsic regulation). Question wording was adapted from Gutentag et al. (2023). Participants responded using a slider scale from 0, *no effort at all*, to 100, *a lot of effort*.

#### Data Analytic Strategy

The analysis plan, analysis codes, de-identified data, and materials for this study can be found at https://osf.io/ydujv/. Analyses were conducted using R (version 4.2.1), on a subset of observations where participants had a significant interaction that day (989 diaries/96% of total diaries completed).

To examine intention frequency, we coded participants’ intention to regulate as 0, *no* and 1, *yes* for intrinsic and extrinsic regulation separately. We also created two additional binary variables to indicate for each survey, whether participants wanted to engage in either kind (i.e., intrinsic = 1 *or* extrinsic = 1), or both kinds (i.e., intrinsic = 1 *and* extrinsic = 1) of regulation. For each of the four binary intention variables, we created a person-mean to reflect the proportion of times a person intended to regulate across the study.

We examined goal and effort data for observations where participants intended to engage in the corresponding regulation type. On the very rare occasion where participants selected an emotion goal and no goal simultaneously, we excluded that survey from analysis, as their response constituted an intention conflict and/or noisy data.

To investigate mean differences in frequencies of intrinsic and extrinsic regulation in multilevel data, we first restructured the data so that for each participant, for each survey, there were two observations: one containing intrinsic intention, and one containing extrinsic intention. We then dummy-coded a categorical variable indicating which observation contained their intrinsic intention response (intention type = intrinsic), and which contained their extrinsic intention response (intention type = extrinsic). Next, we ran a mixed effects multilevel model using the package *lme4* (Bates et al., 2015), where intention type was the predictor, and participants’ intention response was the outcome (Model 1a). The random effects included a random intercept for participants and a random slope for the predictor. The model intercept represented the estimated mean of the reference level, while the slope was the difference between this estimated mean and the other level. For a similar restructuring and modelling procedure, see Neubauer et al., 2020. We employed a similar strategy to examine mean differences in effort levels of intrinsic and extrinsic regulation in Model 1b. Graphical model assumption checks (Fife, 2020) using the package *sjPlot* (Lüdecke et al., 2020) revealed no major violations.

Following helpful comments during review, we post-hoc investigated mean differences in frequencies of goals to only increase positive emotion vs. to only decrease negative emotion. We did not employ the same modelling strategy used in our investigation into mean differences in frequency and effort, because the predictors (i.e., goal type) exhibited multicollinearity. Instead, we calculated person-mean percentage of regulation attempts in which there was a goal to only increase positive emotion vs. to only decrease negative emotion. We then conducted paired *t*-tests to explore whether there was a difference between these person-mean percentages.

### Results and Discussion

Table 1 presents descriptive statistics for all relevant continuous variables. These statistics represented an average across the course of the study, and we observed an effect of time in Study 2, such that the longer participants spent in the study, the more effort they spent turning to others to regulate their own emotions, and the less likely they were to report regulating others’ emotion (see Supplemental Materials A for a full discussion).

**Table 1:** Descriptive statistics for continuous variables

	Study 1 (Daily Diary)				Study 2 (ESM)			
Variable	*M*	*SD*_within_	*SD*_between_	ICC	*M*	*SD*_within_	*SD*_between_	ICC
Any intention	0.54	-	0.33	-	0.44	-	0.27	-
Intrinsic intention	0.29	-	0.31	-	0.25	-	0.23	-
Extrinsic intention	0.47	-	0.32	-	0.36	-	0.26	-
Both intentions	0.19	-	0.25	-	0.17	-	0.20	-
Intrinsic effort	46.01	16.73	21.14	.40	45.20	16.35	17.71	.41
Extrinsic effort	49.96	16.18	21.24	.48	54.65	17.11	17.19	.38

*M* grand mean, *SD*_within_ within-person standard deviation, *SD*_between_ between-person standard deviation, *ICC* intraclass correlation coefficient

#### Primary Analyses

##### How Often do People Intend to Engage in Interpersonal Emotion Regulation?

 Overall, 85% of the sample intended to engage in interpersonal emotion regulation of either the intrinsic or extrinsic type at least once during the study. More people intended to engage in extrinsic regulation than intrinsic regulation, with 8*2*% reporting intending to influence others’ emotions at least once vs. 65% intending to influence their own emotions through others.

At the occasion level, interpersonal emotion regulation of any kind occurred in over half (54%) of participants’ most significant interactions of the day, with intrinsic regulation occurring 29% of the time and extrinsic regulation occurring 47% of the time. Model 1a in Table 2 presents a formal comparison of intrinsic and extrinsic regulation intention frequencies, which revealed intrinsic regulation occurred significantly less often (*p* < .001).

**Table 2:** Comparison of intention and effort level between intrinsic and extrinsic regulation (Study 1)

	Model 1a: intention			Model 1b: effort level		
Predictors	Estimate (*SE*)	95% CI	*p*	Estimate (*SE*)	95% CI	*p*
Intercept: Extrinsic regulation	0.47 (0.02)	0.42 to 0.52	** < .001**	51.33 (1.68)	48.02 to 54.65	** <.001**
Intrinsic regulation	− 0.18 (0.02)	− 0.22 to − 0.13	** < .001**	− 7.14 (1.55)	− 10.18 to − 4.10	** < .001**
N_ID_/observations	168/1,930			145/732		

*SE* standard error, *CI* 95% confidence interval, *N* number of participants

Significant *p*-values bolded. The number of observations had to be doubled during the data restructuring procedure. The effect may be inflated.

In terms of overlap, intrinsic and extrinsic occurred concurrently in around 19% of the interactions recorded. In other words, participants intended to influence both their own and others’ emotions in the same interaction around 19% of the time.

##### What Goals do People Hold?

**Fig. 1:**
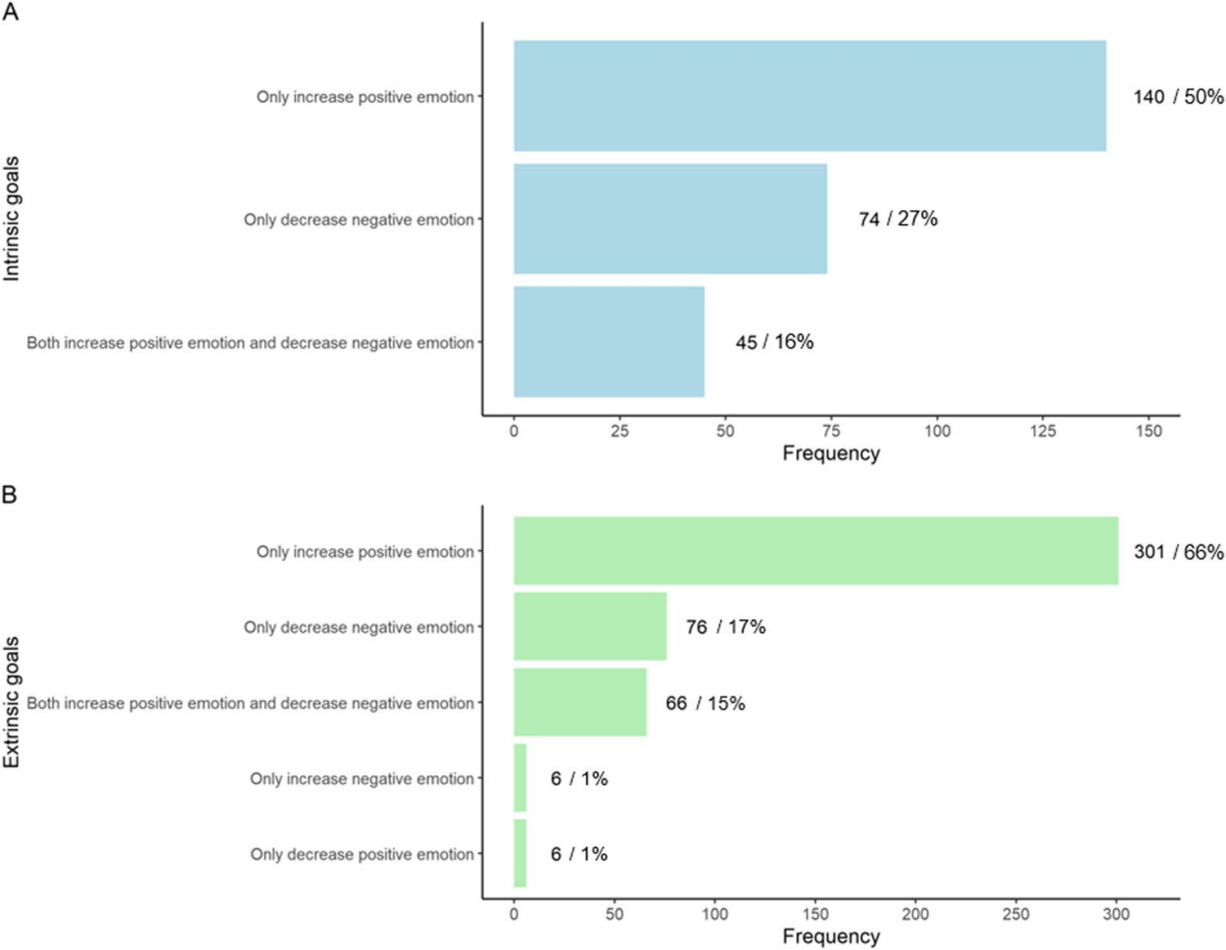
Frequency distribution of **A** intrinsic and **B** extrinsic interpersonal emotion regulation goals (Study 1). *Note.* %, percentage of intrinsic/extrinsic regulation instances with that particular goal. For clarity, low frequency goals are not displayed. For intrinsic regulation, these include goals to both increase and decrease positive emotion (*n* = 5), and to both decrease positive and increase negative emotions (*n* = 1). Goals to only increase negative emotion and to only decrease positive emotion were not reported. For extrinsic regulation, these include goals to increase both positive and negative emotions (*n* = 3), to both increase and decrease positive emotion (*n* = 1), and to decrease both positive and negative emotions (*n* = 1)

 Fig. 1 shows the different goals participants held when they intended to engage in interpersonal emotion regulation. When participants intended to regulate their own emotions through others, 93% of the time it was in service of affect-improving goals, mostly by increasing positive emotions, rather than by decreasing negative emotions or by a combination of increasing positive and decreasing positive emotions. We also observed a similar distribution of goals on occasions when participants intended to regulate others’ emotions, with affect-improving goals being prevalent primarily through increasing positive emotions. Indeed, paired *t*-tests suggested on average, participants had a higher percentage of goals to increase their own positive emotion (*M* = 50.85, *SD* = 41.89) vs. decrease their own negative emotion (*M* = 24.83, *SD* = 35.52); *t*(107) =  − 3.91, *95%* CI =  − 39.21 to − 12.84, *p*   < .001. They also had a higher percentage of goals to increase others’ positive emotion (*M* = 60.37, *SD* = 38.70) vs. decrease others’ negative emotion (*M* = 18.62, *SD* = 31.09); *t*(136) =  − 7.70, *95%* CI =  − 52.47 to − 31.03, *p* <.001.

##### How Much Effort do People Invest?

 The means, within-person standard deviations, between-person standard deviations, and intraclass correlation coefficients (*ICC*) of different effort types are presented in Table 1. Additionally, Model 1b in Table 2 showed that on average, people reported exerting less effort to regulate their own emotions compared to others’ emotions in social interactions (*p* < .001).

#### Supplemental Analyses

Although our main analyses focused on describing the frequency, goals, and effort of interpersonal emotion regulation, we were also interested in examining whether different goals were differentially associated with effort. This exploratory analysis was pre-registered based on prior knowledge of the distribution of emotion goals, such that goals to increase positive emotions were more frequent than goals to decrease negative emotions. As the literature on emotion regulation has primarily focused on the regulation of negative emotions, we were curious as to why positive emotion goals were more prevalent in daily life. Perhaps there were certain characteristics of positive emotion goals that appealed to people, more so than negative emotion goals.

To investigate this question, we ran separate models for intrinsic and extrinsic regulation, investigating whether goals to only increase positive emotion, goals to only decrease negative emotions, and goals to both increase positive and decrease negative emotions, were associated with different effort levels. Full results are in Supplemental Material B.

We found that the goal people had for their own emotions was not associated with how much effort they spent regulating their own emotions through others. In contrast, people put in more effort to regulate others’ emotions when they wanted to both increase positive and decrease negative emotions at the same time (*b* = 14.44, 95% CI = 4.78–24.11, *p* =.003), compared to when they wanted to only increase positive emotion or only decrease negative emotion in others.

#### Discussion

Study 1 revealed that almost everyone engaged in interpersonal emotion regulation at least once throughout the study, although at relatively low frequencies. People also more frequently engaged in, and put more effort towards, regulating others’ emotions, compared to turning to others to regulate their own emotions. Regardless of whether they tried to regulate others’ or their own emotions, the goals were similar: to feel better, primarily through increasing positive rather than decreasing negative emotions. However, to obtain a more accurate reflection of interpersonal emotion regulation across the day would require a method capable of capturing social interactions more frequently than the daily diary method used in Study 1.

## Study 2

In Study 2, we took a more fine-grained approach than in Study 1, with a larger sample using an ESM design, by asking participants to reflect on their most recent social interaction several times a day, and their engagement in interpersonal emotion regulation during the interaction. This approach allowed us to replicate Study 1 findings across a different methodology, as well as to explore a wider range of social interactions across the day.

### Method

#### Participants

We aimed to recruit at least 200 participants, based on an *a priori* power analysis conducted using the *t*-method for multilevel models (Murayama et al., 2022). This sample size allowed us to detect a *t*-value of 2.50 (*d* =.20) with 80% power and *α* = .05.

The final sample comprised 239^2^ participants, aged 18 to 79 (*M* = 29.74, SD = 10.85, 71% women). Recruitment occurred through an undergraduate research participation program, as well as community advertising. Reimbursement for both pools of participants was dependent on their compliance with the ESM protocol.

#### Design and Procedure

The data collection procedure was pre-registered at https://osf.io/5ze6p. It comprised two parts: a baseline survey on Day 1, followed by a 7-day experience sampling period, with seven surveys and one end-of-day survey each day. All variables of interest were in the ESM surveys (see the data collection pre-registration for the full list of measures).

##### Baseline Survey

On Day 1, participants received instructions to download the SEMA3 mobile application (Koval et al., 2019), and a link to the baseline survey on Qualtrics. After providing informed consent and completing a series of trait measures, they then watched videos about the study and the SEMA3 app. Comprehension checks were included to ensure participants understood the ESM protocol.

##### ESM Surveys

The following day, eligible participants began the ESM portion. For seven consecutive days, participants received notifications to complete seven ESM surveys per day, for a total of 49 surveys. We used a mixed sampling scheme, with each survey randomly scheduled within a fixed time window, and each window evenly distributed from 9:30 am to 7:00 pm. Participants had 30 min to complete each survey. ESM surveys occurred 89.98 min apart on average (*SD* = 12.63).

Each ESM survey asked participants to reflect on their most significant social interaction since the previous survey, and on any interpersonal emotion regulation that occurred during the interaction. If participants did not report an interaction, they instead answered questions about their current emotional state, included for even branching. To encourage compliance, participants received email reminders to complete surveys on days 2 and 5 of the ESM period. Overall compliance was 74.49% (*SD* = 19.79). Participants completed on average 36 out of 49 ESM surveys, for a total of 8,678 surveys.

#### Measures

##### Intrinsic and Extrinsic Interpersonal Emotion Regulation Intention and Goal

 To assess regulation intention and goals, we asked participants: “How did you use other people to change your emotions during this interaction?” (intrinsic regulation), and “How did you try to change the emotions of other people during this interaction?” (extrinsic regulation). Participants could select one or more options indicating that they (1) did not try to change their own/others’ emotions, (2) tried to increase or maintain their own/others’ positive emotion, (3) tried to increase or maintain their own/others’ negative emotion, (4) tried to decrease their own/others’ positive emotion, and (5) tried to decrease their own/others’ negative emotion. Selecting only option (1) indicated no intention to regulate, and any of options (2) to (5) indicated an intention to regulate. The decision to measure intention and goal using one item, as opposed to two items in Study 1, was to reduce response burden in the ESM protocol.

##### Intrinsic and Extrinsic Interpersonal Emotion Regulation Effort

 When participants indicated they had a goal for intrinsic and/or extrinsic interpersonal emotion regulation, they were asked how much effort they put into achieving said goal. Question wording was the same as in Study 1.

#### Data Analytic Strategy

The analysis plan, analysis codes, de-identified data, and materials for this study can be found at https://osf.io/pvnas/. Analyses were conducted using R (version 4.1.2), on a subset of observations where participants had a significant interaction since the previous survey (5,534 surveys/64% total surveys completed). To screen for careless responses, we excluded prior to analysis any items completed in less than 650 ms, and any surveys with more than 50% of items responded to in under this time (Geeraerts & Kuppens, 2020). As a result, 233 items (0.8% of all relevant items), and 62 surveys (0.7% of all surveys) were replaced with missing data.

To examine intention frequency, we created two binary variables based on participants’ responses to the intrinsic and extrinsic regulation goal items. Participants’ intention to regulate was coded as 0, *no* and 1, *yes* for intrinsic and extrinsic regulation separately. As in Study 1, we created two additional binary variables to indicate for each survey whether participants intended to engage in either or both types of regulation. We also created person-mean variables for all four intentions.

We analyzed goal and effort variables, as well as mean differences in frequencies, effort levels, and goals to increase positive emotion vs. to decrease negative emotion, of intrinsic and extrinsic regulation, using the same analytic strategies as Study 1.

### Results and Discussion

#### Primary Analyses

##### How Often do People Intend to Engage in Interpersonal Emotion Regulation?

 We found 95% of participants intended to engage in interpersonal emotion regulation of either type at least once during the study. More people intended to engage in extrinsic regulation (92%) than intrinsic regulation (82%).

At the occasion level, 44% of social interactions involved some kind of interpersonal emotion regulation. Intrinsic regulation occurred 25% of the time, and extrinsic regulation 36% of the time. Model 1a in Table 3 showed intrinsic regulation indeed occurred significantly less often than extrinsic regulation (*p* <.001).

**Table 3:** Comparison of intention and effort level between intrinsic and extrinsic regulation (Study 2)

	Model 1a: intention			Model 1b: effort level		
Predictors	Estimate (*SE*)	95% CI	*p*	Estimate (*SE*)	95% CI	*p*
Intercept: extrinsic regulation	0.37 (0.02)	0.34 to 0.40	** < .001**	55.24 (1.08)	53.10 to 57.38	** < .001**
Intrinsic regulation	− 0.12 (0.01)	− 0.15 to − 0.10	** < .001**	− 9.14 (0.80)	− 10.71 to − 7.57	** < .001**
N_ID_/observations	239/10,802			227/3,211		

*SE* standard error, *CI* 95% confidence interval, *N* number of participants

Significant *p*-values bolded. The number of observations had to be doubled during the data restructuring procedure. The effect may be inflated.

In terms of overlap, intrinsic and extrinsic regulation occurred concurrently in 17% of recorded interactions. In other words, participants intended to influence their own emotions through others, and influence others’ emotions, in the same interaction around 17% of the time.

##### What Goals do People Hold?

**Fig. 2:**
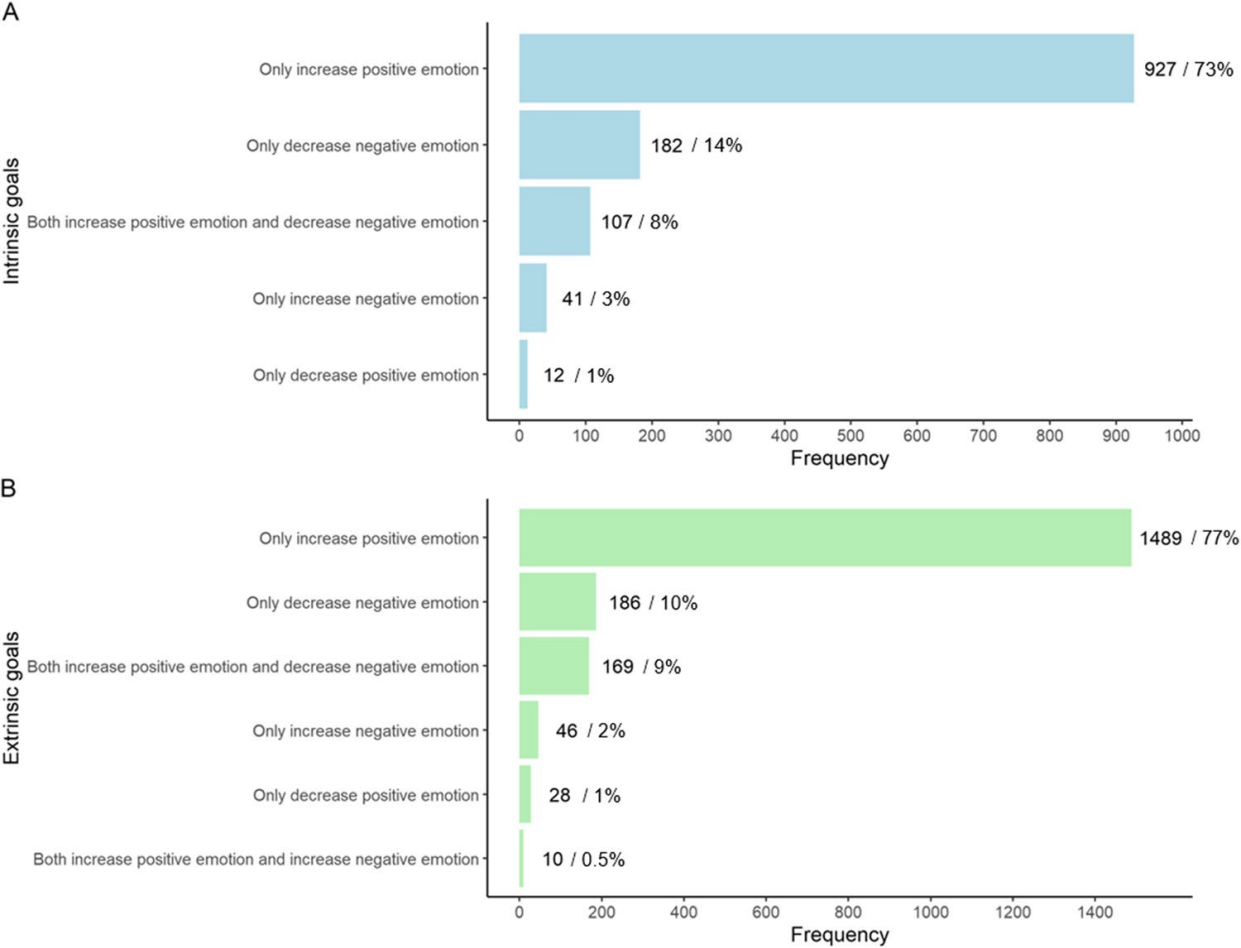
Frequency distribution of **A** intrinsic and **B** extrinsic interpersonal emotion regulation goals (Study 2). *Note.* %, percentage of intrinsic/extrinsic regulation instances with that particular goal. For clarity, low frequency goals are not displayed. For intrinsic regulation, these include goals to both increase and decrease positive emotion (*n* = 5), to increase both positive and negative emotions (*n* = 2), and to decrease both positive and negative emotions (*n* = 2). For extrinsic regulation, these include goals to both decrease positive and increase negative emotions (*n* = 5), to increase positive emotions and both increase and decrease negative emotions (*n* = 4), to both increase and decrease positive emotion (*n* = 3), to both increase and decrease negative emotion (*n* = 2), and to both increase and decrease positive emotion as well as increase and decrease negative emotion (*n* = 1)

 Fig. 2 shows the different goals participants held when they intended to engage in interpersonal emotion regulation. When participants reported an intention to regulate their own emotions through others, or to regulate others’ emotions, they overwhelmingly had goals to only increase positive emotion (intrinsic: 73%, extrinsic: 77%), followed by to only decrease negative emotion (intrinsic: 14%, extrinsic: 10%), and to simultaneously increase positive and decrease negative emotions (intrinsic: 8%, extrinsic: 9%). Results from paired *t*-tests revealed on average, participants had a higher percentage of goals to increase their own positive emotion (*M* = 70.29, *SD* = 31.56) vs. decrease their own negative emotion (*M* = 16.25, *SD* = 25.08); *t*(196) =  − 14.25, 95% CI =  − 61.51 to − 46.55, *p* < .001. They also had a higher percentage of goals to increase others’ positive emotion (*M* = 76.58, *SD* = 26.51) vs. decrease others’ negative emotion (*M* = 10.01, *SD* = 19.58); *t*(220) =  − 23.28, 95% CI =  − 72.12 to − 60.86, *p<* .001.

##### How Much Effort do People Invest?

 Descriptive statistics of both intrinsic and extrinsic regulation effort are presented in Table 1. Model 1b in Table 3 presents a formal comparison between intrinsic and extrinsic effort levels. On average, people reported exerting less effort to regulate their own emotions through others, compared to regulating others’ emotions (*p* < .001).

#### Supplemental Analyses

We also wanted to explore the association between different goals and effort levels in Study 2, using the same data analytic strategy as in Study 1. Full results are in Supplemental Material C. Similar to Study 1, we found the type of goal people had for their own emotions was not associated with how much effort they spent regulating their own emotions through others. In contrast, people put in more effort to regulate others’ emotions when they wanted to decrease others’ negative emotion (*b* = 5.21, 95% CI = 0.48–9.94, *p* = .031), and to simultaneously increase positive and decrease negative emotions of others (*b* = 17.01, 95% CI = 12.14–21.89, *p* < .001), compared to when they only wanted to increase others’ positive emotion.

#### Discussion

Study 2 revealed a similar picture of interpersonal emotion regulation to that in Study 1. Nearly everyone engaged in interpersonal emotion regulation, although they more often—and more effortfully—regulated others’ emotions, compared to regulating their own emotions through others. Regulation goals were almost always to make themselves and others feel better, primarily through increasing positive emotions rather than decreasing negative emotions.

## General Discussion

Using daily life methods, we examined the initiation of intrinsic and extrinsic interpersonal emotion regulation, as well as affect-improving and affect-worsening goals. We found almost everyone engaged in interpersonal emotion regulation, although regulation was by no means a default. While goals to engage in intrinsic and extrinsic regulation were similar, intrinsic regulation was less frequent and less effortful than extrinsic regulation. These findings provide insights into the processes that precede and complement strategy selection and implementation, thereby more comprehensively mapping the interpersonal emotion regulation space.

### How Often do People Intend to Engage in Interpersonal Emotion Regulation?

Supporting prior research (Liu et al., 2021), we found almost everyone regulated at least once during the study. More people regulated others’ emotions (extrinsic) than regulating their own emotions through others (intrinsic), consistent with extant evidence that people more often provide regulatory support than they seek (Reeck et al., 2016). Approximately 36–47% of interactions involved extrinsic regulation vs. 25–29% for intrinsic regulation, vs. 17–19% for both types concurrently. For Study 2, these percentages suggest people regulated others’ emotions nearly twice a day, regulated their own emotions through others around once a day, and regulated their own and others’ emotions in the same interaction roughly every other day. Thus, *inter*personal emotion regulation, particularly intrinsic regulation, appears to be rarer than *intra*personal emotion regulation, which occurs in 43–73% of daily events (English et al., 2017). We note that our findings are based on averages across one week, and future research should investigate interpersonal emotion regulation over a longer period to draw firmer conclusions about its frequencies.

The rarity of intrinsic regulation relative to extrinsic regulation may be due to a few reasons. First, because people reported extrinsic regulation to be more effortful than intrinsic, extrinsic regulation may thus be more noticeable to them, reflecting in the higher frequency reported. Second, since support-seeking can happen unconsciously (Barbee et al., 1993), people may not always be aware they are intrinsically regulating during an interaction. Because we study interpersonal emotion regulation as a conscious process (Niven, 2017; Zaki & Williams, 2013), we captured only *intentional* regulation. Future work might investigate unconscious forms of intrinsic regulation or focus on contexts in which people are more likely to seek support. Nevertheless, given the relatively low frequencies of intentional regulation in everyday life, our findings suggest that researchers investigating strategy use might want to consider assessing intentions first, to ensure they are capturing strategy use on occasions when people actually intend to regulate.

### What Goals do People Hold?

Most intrinsic and extrinsic regulation goals focused on improving affect, primarily by increasing positive emotions. This prevalence was surprising, given existing research often focuses on reducing negative emotions (Webb et al., 2012). Yet, we found when people wanted to make themselves or others feel better, they more often aimed to amplify the positive. It may be that the prompt to reflect on a “significant” interaction could be construed as positive, cueing participants to focus more on positive aspects of the interaction. Another possibility is that striving to feel more positive is an approach goal, whereas avoiding feeling negative is an avoidance goal. While both are central to well-being, approach goals are easier to pursue and monitor (Tamir, 2021), and thus may be more appealing (Tamir & Diener, 2008). Indeed, supplemental analyses found extrinsic goals involving decreasing negative emotions were more effortful. These preliminary findings offer one possible explanation for why negative emotion goals were less frequent: they were more effortful. Nevertheless, this relationship requires a more systematic examination, as it carries implications for effective goal setting and pursuit.

While affect-improving goals were most frequent, people still endorsed affect-worsening goals for themselves and others 3–4% of the time. Research on affect-worsening goals is sparse, likely because they are so rare, either due to people’s unwillingness to report or inability to consciously reflect on such instances (Niven et al., 2011). Yet, we found they do exist in everyday life, and are slightly more frequent for *inter*personal vs. 1.6% for *intra*personal emotion regulation (Kalokerinos et al., 2017). Because goals influence the strategies people choose to achieve that goal (Greenaway et al., 2021; Millgram et al., 2019), investigating affect-worsening goals may help researchers map a more comprehensive strategy repertoire.

### How Much Effort do People Invest?

When people intended to regulate, they invested moderate levels of effort (i.e., 45–55 points on a 100-point scale). They also spent less effort regulating their own emotions through others than regulating others’ emotions. First, it is possible that intrinsic regulation was relatively less effortful because people could draw on intrapersonal *and* interpersonal resources when regulating their own emotions, although how much effort they exert intrapersonally vs. interpersonally remains an interesting unanswered question. Second, merely interacting with others can help reduce negative emotions (Beckes & Coan, 2011). People may benefit from this incidental interpersonal modulation, thereby requiring less conscious effort to seek help regulating their own emotions (Zaki & Williams, 2013).

### Limitations and Future Directions

One notable limitation involves the sampling schedule, which may undermine the accuracy of the frequencies reported. First, because participants could only report one social interaction per survey, our data likely do not capture all interactions people experienced, and thus all regulation instances. Second, because participants completed the surveys after the interaction had already occurred, it is possible that participants may have inferred regulation intention based on interaction outcome. Future work may consider a more temporally fine-grained sampling schedule, or an event-contingent design to capture more frequent (Himmelstein et al., 2019) and emotionally intense interactions critical for regulation processes (Kuppens et al., 2022).

Another limitation concerns the use of single-item measures for intention, goal, and effort in our studies, which reduce participant burden but are possibly prone to measurement error. These measures should be improved by using either a multiple-item validated scale or by including other variables to examine their validity (Kuppens et al., 2022).

Apart from addressing these limitations, future studies can also explore new avenues introduced by the present research. First, contextualizing the social interactions, such as the nature of the interaction, relationship with the interaction partner, or the interaction medium, may provide a better understanding of when interpersonal emotion regulation is most likely to occur. Second, our investigation into effort levels invites a follow-up question of whether this effort pays off. Third, to better understand what is driving regulation initiation, we suggest looking beyond emotion regulation goals, into the higher-order motives these goals serve, as they have implications for how people regulate (Millgram et al., 2019; Tamir et al., 2020). For instance, people may have a goal to increase others’ positive emotions, but this goal may ultimately be in service of a higher-order motive to build social relationships, or to feel better themselves. Research into interpersonal emotion regulation motives is still sparse (cf. Liu et al., 2021; Springstein et al., 2022), with plenty left to explore in what we believe is a worthwhile expedition.

## Conclusion

This research provides insight into the frequency, goals, and effort invested in interpersonal emotion regulation in everyday life. We found almost everyone engaged in interpersonal emotion regulation, although they did not always regulate in every social interaction. While people engaged in intrinsic and extrinsic regulation largely to improve affect, intrinsic regulation was less frequent and less effortful than extrinsic. Our work provides a more nuanced understanding of interpersonal emotion regulation and identifies new avenues to explore how people can regulate more effectively in day-to-day life.

### Supplementary Information

Below is the link to the electronic supplementary material.

Supplementary file1 (DOCX 49 KB)

## Footnotes

1 Fifty-five participants were screened from further participation or excluded prior to analysis for the following reasons (some participants failed multiple criteria): 16 were located outside the specified recruitment location, 22 completed the baseline in less than 10 min which indicated careless responding, 5 formally withdrew, 2 were under 18 years old, 6 did not complete any daily diary, and 17 displayed suspicious bot-like behaviors (e.g., using similar emails, signing up within seconds of one another, and unfeasible geolocation changes with every survey). In addition, 6 participants completed the baseline survey twice, so we kept only the most complete entry.

2 Thirty-two participants were excluded prior to analysis for the following reasons (some participants failed multiple criteria): 9 failed the attention checks, 3 were ineligible to continue to the ESM portion, 1 experienced technical issues, 4 formally withdrew, 6 did not complete any ESM surveys, and 9 had no baseline data. In addition, 5 participants completed the baseline survey twice, so we kept only the most complete entry.

## Additional Information

### Availability of Data and Material

The datasets are available in the Open Science Framework repository at https://osf.io/ydujv/ for Study 1, and at https://osf.io/pvnas/ for Study 2.

### Code Availability

R code used to conduct the analyses is available in the Open Science Framework repository at https://osf.io/ydujv/ for Study 1, and at https://osf.io/pvnas/ for Study 2.
