# Supplementary material for: Mapping Interpersonal Emotion Regulation in Everyday Life
Source: Affect Sci. 2023 Nov 13;4(4):672–83. doi: 10.1007/s42761-023-00223-z (PMC10751271; doi:10.1007/s42761-023-00223-z)
Supplement: Supplementary file 1 — Supplementary file1 (DOCX 49 KB) [file 42761_2023_223_MOESM1_ESM.docx]

**Mapping Interpersonal Emotion Regulation in Everyday Life**

Supplementary Online Material (SOM)

# **Supplemental Material A: Relationship Between Time Spent in the Study and Regulation Intention and Effort**

Research using intensive longitudinal design to assess subjective experiences has long grappled with the effect of repeated assessments over time on people’s self-reported states (Shrout et al., 2017). In addition to the initial elevation bias where time spent in the study are associated with decreased ratings in internal states, there is also the potential for reactivity (i.e., changes to people’s behaviors due to taking part in the study), particularly when assessing behaviors people may not normally pay conscious attention to, such as emotion regulation (Eisele et al., 2023). Given that our studies repeated assessed participants’ intention and effort to engage in interpersonal emotion regulation, we were interested in investigating whether there were any time trends in the data that may indicate an initial elevation bias or measurement reactivity.

## **Data Analytic Strategy**

We conducted additional supplemental analyses for Study 1 (Daily Diary) and Study 2 (ESM), fitting mixed effects models with participants’ time in the study as a predictor of regulation intention and effort. This predictor had a range of 1 to 7 for Study 1, and 1 to 49 for Study 2, representing the first to last surveys participants received, and was a proxy for participants’ time in the study. To quantify the level of evidence for both the presence and absence of effects, we calculated the Bayes Factor for each model and interpreted BF10 following Wetzels et al.’s (2011) guidelines. These guidelines suggest that the data provides decisive evidence for the presence of an effect if BF10>100.

## **Results**

Tables S1 and S2 below present results from analyses examining participants’ time in the study as a predictor of regulation intention and effort across Study 1 and Study 2. While we found little to no evidence that time in the study influenced reports of interpersonal emotion regulation in Study 1, the Bayes Factor provided decisive evidence for the effect of time on extrinsic regulation intention and intrinsic regulation effort in Study 2. Namely, the longer participants spent in the study, the less likely they were to regulate others’ emotion (*OR*=0.99, *95%* *CI*=0.98 – 0.99, *p*<.001) and the more effort they invested in turning to others to regulate their own emotions (*b*=0.22, *95%* *CI*=0.14 – 0.30, *p*<.001). Figure S1 provides a visualization of these time trends.

## **Discussion**

Regarding the increase in intrinsic regulation effort over time, it may be the case that turning to others to regulate their own emotions was something participants hadn’t been consciously aware that they had been doing. Taking part in the study may have prompted participants to monitor their regulation behaviors, resulting in a higher level of conscious intrinsic effort as the study progressed.

Regarding the decreased probability in extrinsic regulation intention over time, because the surveys asked them to reflect on their social interactions, it may be that participants were initially more socially motivated to regulate others' emotions, as they may have perceived that we were assessing their social skills or empathy. As the study progressed, this social desirability bias may have diminished, resulting in the time trend in extrinsic regulation intention observed.

Nevertheless, these explanations remain speculative, based on trends that were found in ad hoc analyses and do not appear across studies. They invite a formal investigation into the frequency and effort of interpersonal emotion regulation over a longer time course.

**Figure S1.**

*The relationship between Survey Number and extrinsic regulation intention and intrinsic regulation effort in Study 2.*


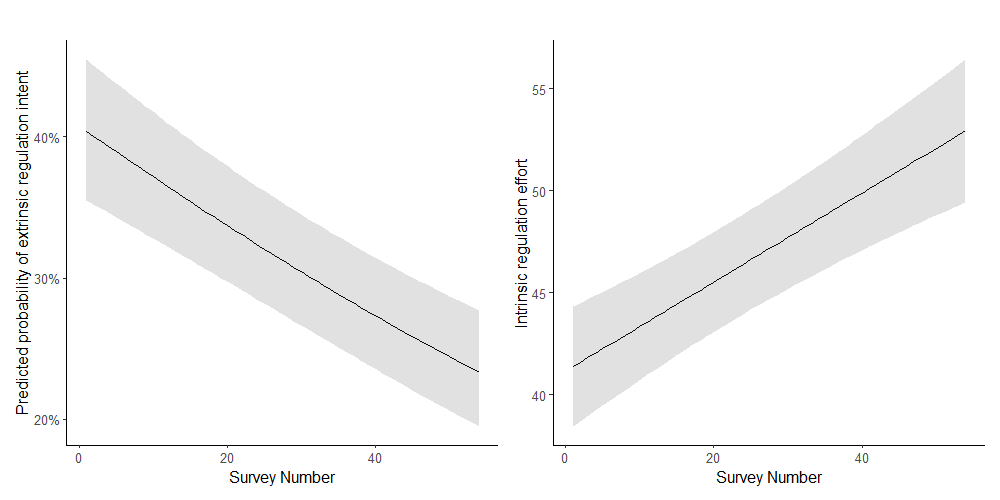


*Note*. Gray band represents 95% confidence interval.

**Table S1.**

*Survey Number as a predictor of intention and effort variables in Study 1.*

|  | **Intrinsic intention** | | | | **Extrinsic intention** | | | | **Intrinsic effort** | | | | | **Extrinsic effort** | | | |
| --- | --- | --- | --- | --- | --- | --- | --- | --- | --- | --- | --- | --- | --- | --- | --- | --- | --- |
| Parameters | *OR (SE)* | *95% CI* | *p* | *BF10* | *OR (SE)* | *95% CI* | *p* | *BF10* | | Estimate (SE) | 95% CI | *p* | *BF10* | Estimate (SE) | 95% CI | *p* | *BF10* |
| Intercept | 0.30 (0.07) | 0.19 – 0.48 | **<.001** | **-** | 1.13 (0.23) | 0.77 – 1.67 | .525 | **-** | | 45.84 (3.15) | 39.64 – 52.03 | **<.001** | **-** | 49.61 (2.53) | 44.63 – 54.59 | **<.001** |  |
| Survey Number | 0.97 (0.04) | 0.89 – 1.06 | .480 | 0.04 | 0.93 (0.03) | 0.86 – 1.00 | **.041** | 0.25 | | -0.03 (0.66) | -1.33 – 1.27 | .966 | 0.03 | 0.38 (0.47) | -0.55 – 1.31 | .425 | 0.07 |
| *N* _ID_ / Observations | 168 / 965 | | | | 167 / 965 | | | | | 108 / 277 | | | | 137/455 | | | |

*Note*. *OR*=Odds ratio; *SE*=Standard error; *CI*=95% confidence interval; *N_ID_*=no. participants; BF10=Bayes Factor in favor of an effect. Significant *p*-values and BF providing decisive evidence for an effect are bolded.

**Table S2.**

*Survey Number as a predictor of intention and effort variables in Study 2.*

|  | **Intrinsic intention** | | | | **Extrinsic intention** | | | | **Intrinsic effort** | | | | | **Extrinsic effort** | | | |  |  |  |  |  |  |  |  |  |  |  |  |
| --- | --- | --- | --- | --- | --- | --- | --- | --- | --- | --- | --- | --- | --- | --- | --- | --- | --- | --- | --- | --- | --- | --- | --- | --- | --- | --- | --- | --- | --- |
| Parameters | *OR (SE)* | *95% CI* | *p* | *BF10* | *OR (SE)* | *95% CI* | *p* | *BF10* | Estimate (SE) | 95% CI | *p* | *BF10* | Estimate (SE) | | 95% CI | *p* | *BF10* |  |  |  |  |  |  |  |  |  |  |  |  |
| Intercept | 0.28 (0.03) | 0.22 – 0.35 | **<.001** | **-** | 0.69 (0.07) | 0.56 – 0.85 | **<.001** | **-** | 41.14  (1.53) | 38.15 – 44.14 | **<.001** | **-** | 53.02  (1.31) | | 50.45 – 55.59 | **<.001** | **-** |  |  |  |  |  |  |  |  |  |  |  |  |
| Survey Number | 0.99 (0.00) | 0.98 – 0.99 | **<.001** | 32.22 | 0.99 (0.00) | 0.98 – 0.99 | **<.001** | **2.98 × 10^7^** | 0.22  (0.04) | 0.14 – 0.30 | **<.001** | **6700.92** | 0.11  (0.03) | | 0.05 – 0.18 | **<.001** | 1.34 |  |  |  |  |  |  |  |  |  |  |  |  |
| *N* _ID_ / Observations | 239 / 5417 | | | | 239 / 5385 | | | | 197 / 1273 | | | | 221 / 1938 | | | | |  |  | 221 / 1938 |  | 197 / 1273 |  |  |  | 221 / 1938 |  |  |  |

*Note*. *OR*=Odds ratio; *SE*=Standard error; *CI*=95% confidence interval; *N_ID_*=no. participants; BF10=Bayes Factor in favor of an effect. Significant *p*-values and BF providing decisive evidence for an effect are bolded.

# **Supplemental Material B: Goals as Predictors of Effort (Study 1)**

Frequency distribution of the different goals people held for intrinsic and extrinsic regulation revealed goals to only increase positive emotions were much more frequent compared to goals to only decrease negative emotions. Because the literature on emotion regulation has primarily focused on the regulation of negative emotions, we were curious as to why positive emotion goals were more prevalent in daily life. Perhaps there was something about positive emotion goals that would appeal to people, more so than negative emotion goals. As such, we were interested in exploring the question of whether the kind of goals people held was associated with the amount of effort they spent in regulation. The analyses were pre-registered at <https://osf.io/4gd7h>.

## **Data Analytic Strategy**

To explore the relationship between goal and effort for both intrinsic and extrinsic regulation, we included only occasions in which participants indicated an intention to regulate, and had goals to improve their own (intrinsic; Model 1a) or others’ (extrinsic; Model 1b) emotion (i.e., upregulating positive emotions and/or downregulating negative emotions). We focused specifically on affect-improving regulation because affect-improving goals tends to be more common in daily life (e.g., Kalokerinos et al., 2017; Springstein et al., 2022), thereby giving our models more power compared to affect-worsening goals. Analyses were conducted using R (version 4.1.2). Using the package *lme4* (Bates et al., 2015), we ran a linear mixed effects model for intrinsic and extrinsic regulation separately, with person-mean centered binary goal variables as predictors, and effort as the outcome variable. In terms of the random effect structure, we included a random intercept for participant, but did not include random slopes for any occasion-level predictors due to convergence issues. Graphical checks for model assumptions (Fife, 2020), using the R package *sjPlot* (Lüdecke, 2020) revealed no major assumption violations.

## **Results**

Table S3 presents the results from the investigation into goal as a predictor of effort for intrinsic regulation (Model 1a) and extrinsic regulation (Model 1b). Because of convergence issues, we had to remove random slopes for all occasion-level predictors. We found the type of goal people had for their own emotions was not associated with how much effort they spent regulating their own emotions through others. In contrast, people put in more effort to regulate others’ emotions when they wanted to both increase positive and decrease negative emotions of others at the same time (*p*=.003).

**Table S3.**

*Association between different types of goal and effort exerted in regulation (Study 1)*

|  | **Model 1a: Intrinsic effort** | | | **Model 1b: Extrinsic effort** | | |  |
| --- | --- | --- | --- | --- | --- | --- | --- |
| *Predictors* | *Estimate (SE)* | *95% CI* | *p* | *Estimate (SE)* | *95% CI* | *p* | |
| Intercept | 46.30 (3.10) | 40.19 – 52.42 | **<.001** | 48.39 (2.34) | 43.77 – 53.00 | **<.001** | |
| Increase positive emotion | -3.86 (5.49) | -14.68 – 6.95 | .482 | 4.94 (4.27) | -3.46 – 13.34 | .248 | |
| Decrease negative emotion | 3.40 (5.75) | -7.91 – 14.71 | .555 | 9.23 (4.70) | -0.01 – 18.47 | .050 | |
| Both increase positive and decrease negative emotions | -0.00 (6.26) | -12.33 – 12.33 | 1.000 | 14.44 (4.92) | 4.78 – 24.11 | **.003** | |
| N _ID /_ Observations | 108 / 277 | | | 137 / 455 | | |  |

*Note*. *SE* = Standard error; *CI* = 95% confidence interval; *N* = number of participants. Significant *p*-values bolded.

# **Supplemental Material C: Goals as Predictors of Effort (Study 2)**

## **Data Analytic Strategy**

To explore the relationship between goal and effort for both intrinsic and extrinsic regulation, we employed the same modelling technique as in Study 1. Graphical checks for model assumptions (Fife, 2020) revealed no major assumption violations.

In a departure from our pre-registered models (<https://osf.io/4jzwp>), we did not include the effort at the previous survey (i.e., lagged variable) as a control variable. This decision was because lagging variables relied on surveys being consecutive. However, consecutive surveys are not always the case in ESM data in which participants sometimes skip surveys (Sun et al., 2021), and this lack is more pronounced in our data, because we only examined regulation in observations where people reported having a social interaction *and* an intention to regulate. These inclusion criteria resulted in many surveys being excluded from lagged analyses, thereby reducing our power (e.g., there were 429 available observations to examine the relationship between extrinsic goals and extrinsic effort when we included a lagged control vs. 1,938 observations when we did not).

## **Results**

Models 2a and 2b in Table S4 examined the association between different regulation goals and effort levels. Because of convergence issues, we had to remove random slopes for all occasion-level predictors. Similar to Study 1, we found the type of goal people had for their own emotions was not associated with how much effort they spent regulating their own emotions through others. In contrast, people put in more effort to regulate others’ emotions when they wanted to decrease others’ negative emotion (*p*=.031), and to simultaneously increase positive and decrease negative emotions of others (*p*<.001).

**Table S4.**

*Association between different types of goal and effort exerted in regulation (Study 2)*

|  | **Model 2a: Intrinsic effort (unlagged)** | | | **Model 2b: Extrinsic effort (unlagged)** | | |  |
| --- | --- | --- | --- | --- | --- | --- | --- |
| *Predictors* | *Estimate (SE)* | *95% CI* | *p* | *Estimate (SE)* | *95% CI* | *p* | |
| Intercept | 46.66 (2.00) | 42.74 – 50.59 | **<.001** | 53.05 (1.53) | 50.04 – 56.06 | **<.001** | |
| Increase positive emotion | -2.25 (2.50) | -7.16 – 2.66 | .368 | 3.18 (1.97) | -0.68 – 7.03 | .106 | |
| Decrease negative emotion | 0.62 (2.90) | -5.06 – 6.30 | .830 | 5.21 (2.41) | 0.48 – 9.94 | **.031** | |
| Both increase positive and decrease negative emotions | 5.45 (3.19) | -0.81 – 11.72 | .088 | 17.01 (2.49) | 12.14 – 21.89 | **<.001** | |
| N _ID /_ Observations | 197 / 1,273 | | | 221/ 1,938 | | |  |

*Note*. *SE* = Standard error; *CI* = 95% confidence interval; *N* = number of participants. Significant *p*-values bolded.

To test the robustness of these models, we conducted the original pre-registered analyses where we controlled for lagged effort levels (Table S5). We found one of the effects in Model 2b remained significant when we included the lagged control, such that people put in more effort to regulate others’ emotions only when they wanted to simultaneously increase positive and decrease others’ negative emotions (*p*=.038). We note, however, that due to the low number of observations available for lagged analysis, it is possible that the lagged model did not have enough power to detect an effect.

**Table S5.**

*Association between different types of goal and effort exerted in regulation, controlling for previous effort levels (Study 2)*

|  | **Model 2a: Intrinsic effort (lagged)** | | | **Model 2b: Extrinsic effort (lagged)** | | |  |
| --- | --- | --- | --- | --- | --- | --- | --- |
| *Predictors* | *Estimate (SE)* | *95% CI* | *p* | *Estimate (SE)* | *95% CI* | *p* | |
| Intercept | 50.93 (3.71) | 43.60 – 58.26 | **<.001** | 54.79 (2.73) | 49.41 – 60.17 | **<.001** | |
| Increase positive emotion | -3.08 (5.85) | -14.60 – 8.44 | .599 | 1.59 (4.82) | -7.90 – 11.07 | .742 | |
| Decrease negative emotion | 1.56 (6.60) | -11.44 – 14.56 | .813 | 4.83 (5.88) | -6.72 – 16.38 | .411 | |
| Both increase positive and decrease negative emotions | 13.95 (7.53) | -0.89 – 28.79 | .065 | 12.81 (6.14) | 0.73 – 24.88 | **.038** | |
| Previous effort level | 0.27 (0.10) | 0.08 – 0.47 | **.008** | 0.13 (0.06) | 0.01 – 0.25 | **.038** | |
| N _ID /_ Observations | 83 / 245 | | | 127 / 429 | | |  |

*Note*. *SE* = Standard error; *CI* = 95% confidence interval; *N* = number of participants. Significant *p*-values bolded.

# **References**

Bates, D., Kliegl, R., Vasishth, S., & Baayen, R. H. (2015). Parsimonious Mixed Models. *arXiv*. <https://doi.org/10.48550/arXiv.1506.04967>

Eisele, G., Vachon, H., Lafit, G., Tuyaerts, D., Houben, M., Kuppens, P., Myin-Germeys, I., & Viechtbauer, W. (2023). A mixed-method investigation into measurement reactivity to the experience sampling method: The role of sampling protocol and individual characteristics. *Psychological Assessment, 35*(1), 68–81. [https://doi.org/10.1037/pas0001177](https://psycnet.apa.org/doi/10.1037/pas0001177)

Fife, D. (2020). The Eight Steps of Data Analysis: A Graphical Framework to Promote Sound Statistical Analysis. *Perspectives on Psychological Science*, *15*(4), 1054–1075. <https://doi.org/https://doi.org/10.1177/174569162091733>

Kalokerinos, E. K., Tamir, M., & Kuppens, P. (2017). Instrumental motives in negative emotion regulation in daily life: Frequency, consistency, and predictors. *Emotion, 17*(4), 648–657. <https://doi.org/10.1037/emo0000269>

Lüdecke, D., Ben-Shachar, M., Patil, I., & Makowski, D. (2020). Extracting, computing and exploring the parameters of statistical models using R. *Journal of Open Source Software*, *5*(53), 2445. <https://doi.org/10.21105/joss.02445>

Shrout, P. E., Stadler, G., Lane, S. P., McClure, M. J., Jackson, G. L., Clavél, F. D., Iida, M., Gleason, M. E. J., Xu, J. H., & Bolger, N. (2018). Initial elevation bias in subjective reports. *PNAS Proceedings of the National Academy of Sciences of the United States of America, 115*(1), E15–E23. [https://doi.org/10.1073/pnas.1712277115](https://psycnet.apa.org/doi/10.1073/pnas.1712277115)

Springstein, T., Hamerling-Potts, K. K., Landa, I., & English, T. (2022). Adult attachment and interpersonal emotion regulation motives in daily life. *Emotion*. <https://doi.org/10.1037/emo0001169>

Sun, J., Rhemtulla, M., & Vazire, S. (2021). Eavesdropping on Missing Data: What Are University Students Doing When They Miss Experience Sampling Reports?. *Personality & social psychology bulletin*, *47*(11), 1535–1549. <https://doi.org/10.1177/0146167220964639>

Wetzels, R., Matzke, D., Lee, M. D., Rouder, J. N., Iverson, G. J., & Wagenmakers, E.-J. (2011). Statistical Evidence in Experimental Psychology: An Empirical Comparison Using 855 t Tests. *Perspectives on Psychological Science, 6*(3), 291–298. <https://doi.org/10.1177/1745691611406923>
